# GWAS Analysis and QTL Identification of Fiber Quality Traits and Yield Components in Upland Cotton Using Enriched High-Density SNP Markers

**DOI:** 10.3389/fpls.2018.01067

**Published:** 2018-09-13

**Authors:** Ruixian Liu, Juwu Gong, Xianghui Xiao, Zhen Zhang, Junwen Li, Aiying Liu, Quanwei Lu, Haihong Shang, Yuzhen Shi, Qun Ge, Muhammad S. Iqbal, Xiaoying Deng, Shaoqi Li, Jingtao Pan, Li Duan, Qi Zhang, Xiao Jiang, Xianyan Zou, Abdul Hafeez, Quanjia Chen, Hongwei Geng, Wankui Gong, Youlu Yuan

**Affiliations:** ^1^Xinjiang Research Base, State Key Laboratory of Cotton Biology, Xinjiang Agricultural University, Urumqi, China; ^2^State Key Laboratory of Cotton Biology, Institute of Cotton Research, Chinese Academy of Agricultural Sciences, Anyang, China; ^3^School of Biotechnology and Food Engineering, Anyang Institute of Technology, Anyang, China

**Keywords:** upland cotton, QTL, multilocus GWAS, QTN, candidate gene, fiber quality traits, yield components

## Abstract

It is of great importance to identify quantitative trait loci (QTL) controlling fiber quality traits and yield components for future marker-assisted selection (MAS) and candidate gene function identifications. In this study, two kinds of traits in 231 F_6:8_ recombinant inbred lines (RILs), derived from an intraspecific cross between Xinluzao24, a cultivar with elite fiber quality, and Lumianyan28, a cultivar with wide adaptability and high yield potential, were measured in nine environments. This RIL population was genotyped by 122 SSR and 4729 SNP markers, which were also used to construct the genetic map. The map covered 2477.99 cM of *hirsutum* genome, with an average marker interval of 0.51 cM between adjacent markers. As a result, a total of 134 QTLs for fiber quality traits and 122 QTLs for yield components were detected, with 2.18–24.45 and 1.68–28.27% proportions of the phenotypic variance explained by each QTL, respectively. Among these QTLs, 57 were detected in at least two environments, named stable QTLs. A total of 209 and 139 quantitative trait nucleotides (QTNs) were associated with fiber quality traits and yield components by four multilocus genome-wide association studies methods, respectively. Among these QTNs, 74 were detected by at least two algorithms or in two environments. The candidate genes harbored by 57 stable QTLs were compared with the ones associated with QTN, and 35 common candidate genes were found. Among these common candidate genes, four were possibly “pleiotropic.” This study provided important information for MAS and candidate gene functional studies.

## Introduction

Cotton is an important cash crop that provides major natural fiber supply for textile industry and human daily life. Four species in *Gossypium*, namely *G. herbaceum* (A1), *G. arboreum* (A2), *G. hirsutum* (AD1), and *G. barbadense* (AD2), are cultivated ones. *G. hirsutum* (2*n* = 4*x* = 52, genome size: 2.5 Gb) (Li et al., 2014, 2015; Wendel and Grover, 2015; Zhang et al., 2015), also called upland cotton, has a high yield potential, whereas fair fiber quality attributes (Cai et al., 2014), thus making it most widely cultivated and utilized worldwide, approximately accounting for 95% of global cotton fiber production (Chen et al., 2007). Along with the progress of technologies in textile industry and improvement of human living standard, the demand for cotton fiber supply not only increases in quantity but also is required in a diverse combination of various qualities such as high strength, natural color, various lengths, and fineness. Fiber quality traits and yield components are quantitative and controlled by multiple genes (Said et al., 2013), yet most of which were negatively correlated with each other (Shen et al., 2007; Wang H. et al., 2015). Therefore, it is difficult to improve all these traits simultaneously by traditional breeding programs, even after time-consuming and laborious efforts were put (Shen et al., 2005; Lacape et al., 2009; Jamshed et al., 2016; Zhang et al., 2016). The rapid development of applied genome research provides an effective tool for improving plant breeding efficiency, a typical example of which is the marker-assisted selection (MAS) and genome selection through the molecular markers closely linked to target genes or quantitative trait loci (QTLs).

Currently, plenty of intraspecific segregating populations of *G. hirsutum* are constructed targeting various traits in upland cotton, and many QTLs are identified, including those for fiber quality traits (Shen et al., 2005; Sun F.D. et al., 2012; Fang et al., 2014; Xu et al., 2014; Tan et al., 2015; Wang H. et al., 2015; Jamshed et al., 2016; Li et al., 2016; Yang et al., 2016; Liu et al., 2017; Zhang Z. et al., 2017), yield components (Xia et al., 2014; Wang H. et al., 2015; Zhang et al., 2016; Liu et al., 2017), drought tolerances (Levi et al., 2011), disease resistances (Jiang et al., 2009; Ulloa et al., 2013; Zhao et al., 2014; Palanga et al., 2017), early maturity (Stiller et al., 2004; Li et al., 2012, 2013), and plant morphological traits (Tang and Xiao, 2014; Qi et al., 2017).

A genome-wide association studies (GWAS) is also an effective approach for connecting phenotypes and genotypes in plants, and helps us to avoid the difficulty of screening large biparental mapping populations, so it is widely applied to various studies (Thornsberry et al., 2001; Flint-Garcia et al., 2005; Maccaferri et al., 2005; Eizenga et al., 2006; Zhu et al., 2008; Jia et al., 2014; Nie et al., 2016) to identify quantitative trait nucleotides (QTNs) for complex traits (Zhao et al., 2011; Fernandes et al., 2012; Segura et al., 2012; Spindel et al., 2015). It has been successfully applied to *Arabidopsis thaliana* (Atwell et al., 2010; Horton et al., 2012), rice (Huang et al., 2010; Zhao et al., 2011), corn (Kump et al., 2011; Samayoa et al., 2015), and soybean (Dhanapal et al., 2015; Zeng et al., 2017), and many QTNs and their candidate genes have been identified for various ecological and agricultural traits. More recently, it has also been used in cotton (Abdurakhmonov et al., 2008; Kantartzi and Stewart, 2008; Zeng et al., 2009; Cai et al., 2014; Mei et al., 2013; Zhang et al., 2013; Su et al., 2016; Huang et al., 2017; Sun et al., 2017). To better understand the genetic architecture of fiber quality traits and yield components in upland cotton, we genotyped an intraspecific recombinant inbred lines (RILs) using enriched high-density markers of both single-nucleotide polymorphisms (SNPs) based on the CottonSNP80K arrays (Cai et al., 2017) and simple sequence repeats (SSRs). To obtain reliable QTLs and their candidate genes, we tried to use two strategies. One was linkage-map-based QTL mapping, in which a high coverage genetic linkage map was constructed with HighMap software and QTLs were mapped using composite interval mapping (CIM); the other was GWAS along with four multilocus GWAS methods (Wang et al., 2016; Tamba et al., 2017; Wen et al., 2017; Zhang J. et al., 2017). The results in the study could be worthy for further studies not only in molecular-assisted breeding through MAS but also in functional gene validations, which is of great significance to the improvement of cotton fiber quality and yield.

## Materials and Methods

### Plant Materials

An RIL population of 231 lines was developed from a cross between two homozygous upland cotton cultivars, Lumianyan28 (LMY28), a commercial transgenic cultivar with high yield potential and wide adaptability developed by the Cotton Research Center of Shandong Academy of Agricultural Sciences as a maternal line, and Xinluzao24 (XLZ24), a high fiber quality upland cotton cultivar with long-staple developed by XinJiang KangDi company as a paternal line.

The RIL development was briefed as follows: the cross between LMY28 and XLZ24 was made in the summer growing season in 2008 in Anyang, Henan Province. F_1_ were planted and self-pollinated in the winter growing season in 2008 in Hainan Province. In the spring of 2009, 238 F_2_ plants were grown and self-pollinated, and F_2:3_ seeds were harvested in Anyang (Kong et al., 2011). Of the 238 F_2:3_ lines, 231 were self-pollinated in each generation until F_2:6_. Then single plant selection was made from each of the 231 F_2:6_ lines to form the F_6:7_ population. The F_6:7_ population was planted in plant rows and self-pollinated to construct the F_6:8_ RIL population. All the generations beyond F_6:8_ are regarded as F_6:8_ for convenience of analysis. The target traits of the F_6:8_ RIL population were evaluated in Henan (Anyang, 2013, 2014, 2015, and 2016, designated as 13AY, 14AY, 15AY, and 16AY, respectively), Shandong (LinQing, 2013 and 2014, designated as 13LQ and 14LQ, respectively), Hebei (Quzhou 2013, designated as 13QZ), and Xinjiang (Kuerle 2014 and Alaer 2015, designated as 14KEL and 15ALE, respectively), and a randomized complete block design with two replications was adopted in all nine environmental evaluations. A single-row plot with 5-m row length, 0.8-m row spacing, and 0.25-m plant spacing was adopted in 13AY, 13LQ, 13QZ, 14AY, 14LQ, 15AY, and 16AY, whereas a two-narrow-row plot with 3-m row length, 0.66/0.10-m alternating row spacing, and 0.12-m plant spacing were adopted in 14KEL and 15ALE.

### Phenotypic Detection and Data Analysis

Thirty naturally opened bolls from each plot were hand-harvested on the inner fruiting nods from middle to upper branches. Yield component traits, including boll weight (BW, g), lint percentage (LP, %), and seed index (SI, g), were evaluated. No less than 15 g fibers were sampled to evaluate the fiber quality traits, including fiber length (FL, mm), fiber strength (FS, cN tex^-1^), and fiber micronaire (FM). The evaluations were conducted using HFT9000 (Premier Evolvics Pvt. Ltd., India) instruments with HVICC Calibration in the Cotton Quality Supervision, Inspection and Testing Center, Ministry of Agriculture, Anyang, Henan Province, China.

One-way analysis of variance (ANOVA) between parents and the descriptive statistics for the RIL population was conducted using Microsoft Excel 2016, and correlation analysis was performed using SPSS 20.0 (SPSS, Chicago, IL, United States). Integrated ANOVA across nine environments along with the heritability of all the traits was conducted using ANOVA function in the QTL IciMapping software.

### DNA Extraction and Genotyping

Genomic DNA was extracted from fresh leaves of parents and 231 RILs with a modified cetyltrimethyl ammonium bromide (CTAB) method (Song et al., 1998). The DNA was used both for SSR screening and CottonSNP80K array hybridization.

A total of 9668 pairs of SSR primer pool, which contained a variety of sources including NAU, BNL, DPL, CGR, PGML, SWU, and CCRI, were used to screen the polymorphisms between parents. The primer information was also available at the CottonGen Database^1^. PCR amplification and product detection were conducted according to the procedures described by Zhang et al. (2005). The polymorphic primers between the parents were used to genotype the population, and the SSR markers that were codominant and had a unique physical location in the reference genome were used to construct the linkage map.

The cottonSNP80K array, which contained 77,774 SNPs (Cai et al., 2017), was used to genotype the parents and the 231 RILs. The genotyping was conducted according to the Illumina suggestions (Illumina Inc., San Diego, CA, United States) (Cai et al., 2017). After genotyping, the raw data were filtered based on the following criteria (Zhang Z. et al., 2017): first, any or both of the SNP loci of parents were missing (69,395 SNPs were remained after filtering); second, the loci had no polymorphism between parents (15,128 loci were remained); third, the loci of any of the parent were heterozygous (7480 SNPs were remained); forth, the missing rate of SNPs in the population was more than 40% (Hulse-Kemp et al., 2015) (7479 loci were remained); and finally, the segregation distortion of SNPs reached criteria of *P* < 0.001 (5202 loci were remained). Subsequently, the remaining SNP markers were applied to the genetic map construction after converting into the “ABH” data format as SSR.

### Genetic Map Construction

The remaining SSR and SNP markers were divided into the 26 chromosomes based on their position on the physical map of the upland cotton (TM-1) genome database (Zhang et al., 2015). Then, the genetic linkage map was constructed using the HighMap software with multiple sorting and error-correcting functions (Liu et al., 2014). Map distances were estimated using Kosambi’s mapping function (Kosambi, 1943).

The significance of segregation distortion markers (SDMs; *P* < 0.05) was detected using the chi-square test. The regions containing at least three consecutive SDMs were defined as segregation distortion regions (SDRs) (Zhang et al., 2016). The distribution of SDMs and SDRs, and the size of SDRs on the map were analyzed.

### QTL Mapping and Genome-Wide Association Studies

The Windows QTL Cartographer 2.5 software (Wang et al., 2012) was employed using the CIM method with a mapping step of 1.0 cM and five control markers (Zeng, 1994) for QTL identification. The threshold value of the logarithm of odds (LOD) was calculated by 1000 permutations at the 0.05 significance level. QTLs, identified in different environments and had fully or partially overlapping confidence intervals, were regarded as the same QTL. The QTL detected in at least two environments was regarded as a stable one. Nomenclature of QTL was designated following Sun’s description (Sun F.D. et al., 2012). MapChart 2.3 (Voorrips, 2002) was used to graphically represent the genetic map and QTL.

Quantitative trait nucleotides for the target traits were identified by four multilocus GWAS methods. The first one is mrMLM (Wang et al., 2016), in which calculate Kinship (K) matrix model was used, with critical *P*-value of 0.01, search radius of the candidate gene of 20 kb, and critical LOD score for significant QTN of 3. The second one is FASTmrEMMA (Wen et al., 2017), with restricted maximum likelihood, in which calculate K matrix model was used, critical *P-*value of 0.005, and critical LOD score for significant QTN of 3. The third one is ISIS EM-BLASSO (Tamba et al., 2017), with critical *P-*value of 0.01. The fourth one is pLARmEB (Zhang J. et al., 2017); each chromosome selected 50 potential associations at a critical LOD score of 2 with variable selection through LAR.

### QTL Congruency Comparison With Previous Studies

Previous QTLs for the target traits were detected and downloaded in the CottonQTLdb database^2^ (Said et al., 2015). The QTLs sharing similar genetic positions (spacing distance < 15 cM) were regarded as common or same QTL. The physical positions of a QTL were identified in the CottonGen database^3^. When a QTL in the current study shared the same physical region as the previous QTL, it was regarded as a repeated identification of the previous QTL; otherwise, the QTL in the current study was regarded as a new one.

### The Candidate Genes Identification

Candidate genes harbored in the stable QTLs were searched and identified based on their confidence intervals in the following steps: The markers including the closest flanking ones in the confidence interval of a QTL were identified. The physical interval of that QTL was determined based on the physical position of its markers in the upland cotton (TM-1) genome^4^ (Zhang et al., 2015). All the genes in the physical interval were identified as candidate genes.

Candidate genes associated with QTNs in the multilocus GWAS analysis were confirmed based on the location of QTNs in the upland cotton (TM-1) reference genome (Zhang et al., 2015). The gene in which the QTL was located was considered as the candidate gene. But when the physical location of a QTN was between two genes, both of the genes were considered as candidate genes.

## Results

### Phenotypic Evaluation of the RIL Populations

The one-way ANOVA between parents in nine environments showed that a significant difference for FS at the 0.001 level and no significant differences for the other traits were observed (**Table 1**). The descriptive statistical analysis showed that all traits in the RIL population performed transgressive segregations, with approximately normal distribution in all the nine environments (**Table 1**). The integrated ANOVA of the RILs across nine environments also revealed significant variations for all traits among the RILs (**Supplementary Table S1**).

**Table 1:** The results of the statistical analysis of the parents and the RIL population.

Trait^a^	Env.^b^	Parents				RIL population							
		XLZ24^c^	LMY28^d^	Range	*P*-value	Min.	Max.	Range	Mean	*SD*	Var.	Skew.	Kurt.
FL	13AY	29.72	29.23	0.49	0.06	28.12	32.52	4.40	30.54	0.92	0.03	–0.13	–0.44
	13LQ	30.85	29.57	1.28		27.86	33.05	5.19	30.31	1.02	0.03	0.28	–0.10
	13QZ	29.04	28.28	0.75		27.29	33.61	6.32	29.60	1.16	0.04	0.52	0.72
	14AY	31.43	29.44	2.00		28.85	33.65	4.80	31.26	1.00	0.03	0.11	–0.36
	14KEL	29.77	29.69	0.09		28.35	34.07	5.72	31.14	1.04	0.03	0.35	0.23
	14LQ	30.80	30.40	0.40		28.90	34.05	5.15	31.27	1.04	0.03	0.15	–0.30
	15AY	29.95	26.85	3.10		26.15	31.60	5.45	29.06	1.06	0.04	–0.12	–0.35
	15ALE	29.10	29.20	–0.10		26.60	31.80	5.20	29.05	1.08	0.04	0.22	–0.31
	16AY	28.95	28.80	0.15		28.40	34.05	5.65	30.55	0.99	0.03	0.61	1.02
FS	13AY	33.95	30.45	3.50	<0.001***	29.15	39.30	10.15	33.39	1.90	0.06	0.44	–0.03
	13LQ	30.15	27.55	2.60		27.20	36.25	9.05	32.14	1.82	0.06	0.01	–0.26
	13QZ	31.65	29.20	2.45		28.10	39.10	11.00	32.69	2.05	0.06	0.43	–0.16
	14AY	31.95	29.45	2.50		28.55	36.85	8.30	32.42	1.55	0.05	0.18	0.01
	14KEL	31.55	29.15	2.40		27.65	37.70	10.05	32.02	1.75	0.05	0.46	0.49
	14LQ	30.40	29.10	1.30		28.80	37.50	8.70	32.77	1.60	0.05	0.36	0.23
	15AY	33.75	28.60	5.15		27.85	39.65	11.80	33.48	2.10	0.06	0.09	0.21
	15ALE	31.25	27.90	3.35		26.90	35.15	8.25	30.92	1.64	0.05	0.07	–0.14
	16AY	32.80	27.85	4.95		28.75	38.05	9.30	32.98	1.67	0.05	0.21	0.08
FM	13AY	4.49	4.48	0.00	0.46	2.96	5.42	2.46	4.26	0.50	0.12	–0.08	–0.42
	13LQ	3.91	4.41	–0.50		2.25	5.32	3.07	3.91	0.59	0.15	–0.31	–0.15
	13QZ	5.09	4.70	0.40		2.74	5.58	2.84	4.29	0.61	0.14	–0.43	–0.36
	14AY	4.79	4.65	0.14		3.50	5.62	2.12	4.59	0.40	0.09	–0.12	–0.21
	14KEL	4.58	4.56	0.02		3.56	5.28	1.72	4.43	0.34	0.08	0.04	–0.17
	14LQ	5.30	4.85	0.45		3.60	5.50	1.90	4.74	0.38	0.08	–0.36	–0.11
	15AY	4.65	4.95	–0.30		3.50	5.60	2.10	4.55	0.39	0.09	–0.12	–0.20
	15ALE	4.60	4.30	0.30		4.00	5.55	1.55	4.86	0.32	0.07	–0.20	–0.60
	16AY	5.30	4.70	0.60		3.65	5.80	2.15	4.77	0.41	0.09	–0.22	–0.06
BW	13AY	5.47	5.79	–0.32	0.16	4.40	7.20	2.80	5.93	0.50	0.08	–0.29	0.34
	13LQ	5.26	5.58	–0.33		3.76	7.07	3.31	5.55	0.69	0.12	–0.27	–0.57
	13QZ	5.03	5.61	–0.58		3.12	6.87	3.75	5.11	0.77	0.15	–0.24	–0.40
	14AY	5.68	6.13	–0.46		5.24	7.88	2.64	6.39	0.50	0.08	0.16	0.00
	14KEL	6.04	6.16	–0.12		4.65	7.41	2.76	6.23	0.53	0.09	–0.21	0.19
	14LQ	6.23	6.42	–0.19		4.80	8.12	3.32	6.80	0.62	0.09	–0.49	0.32
	15AY	5.44	5.65	–0.21		4.21	6.69	2.48	5.41	0.42	0.08	0.05	0.65
	15ALE	4.91	5.00	–0.08		4.97	7.22	2.25	5.99	0.43	0.07	0.20	–0.21
	16AY	5.47	5.81	–0.34		4.62	7.81	3.19	6.27	0.54	0.09	–0.08	0.28
LP	13AY	41.87	39.01	2.86	0.15	29.56	43.56	14.00	37.82	2.33	0.06	–0.45	0.88
	13LQ	38.53	36.95	1.59		29.02	42.59	13.57	36.36	2.38	0.07	–0.02	0.13
	13QZ	37.93	36.52	1.41		27.09	42.97	15.88	35.61	3.06	0.09	–0.22	0.09
	14AY	44.48	42.13	2.35		35.70	46.73	11.03	41.16	1.99	0.05	–0.15	0.00
	14KEL	43.07	39.10	3.97		33.34	46.03	12.69	39.62	2.21	0.06	–0.10	0.08
	14LQ	42.74	42.42	0.32		33.96	48.51	14.55	41.14	2.39	0.06	–0.14	0.34
	15AY	47.08	44.53	2.55		31.56	47.15	15.59	40.80	2.42	0.06	–0.58	1.69
	15ALE	43.42	41.96	1.46		38.10	48.66	10.56	44.33	1.92	0.04	–0.49	0.53
	16AY	41.59	40.24	1.36		34.09	46.83	12.74	39.49	2.30	0.06	0.29	0.04
SI	13AY	10.60	11.00	–0.40	0.68	9.39	14.44	5.05	11.80	1.03	0.09	0.11	–0.31
	13LQ	10.66	11.63	–0.97		9.23	14.18	4.95	11.84	1.05	0.09	0.22	–0.43
	13QZ	12.2	11.48	0.76		9.31	15.20	5.89	12.21	1.17	0.10	0.11	–0.05
	14AY	–	–	–		–	–	–	–	–	–	–	–
	14KEL	11.53	11.97	–0.45		10.09	14.80	4.71	12.28	1.08	0.09	0.21	–0.76
	14LQ	–	–	–		–	–	–	–	–	–	–	–
	15AY	9.55	9.85	–0.30		8.25	12.55	4.30	10.26	0.84	0.08	0.19	–0.24
	15ALE	9.92	9.77	0.15		8.73	13.40	4.67	10.47	0.89	0.09	0.45	0.06
	16AY	12.55	12.13	0.43		10.13	15.35	5.22	12.55	1.01	0.08	0.24	0.03

*^*a*^FL, fiber length; FS, fibers strength; FM, fiber micronaire; BW, weight; LP, lint percent; SI, seed index. ^*b*^13AY, Anyang in 2013; 13LQ, Linqing in 2013; 13QZ, Quzhou in 2013; 14AY, Anyang in 2014; 14KEL, Kerle in 2014; 14LQ, Linqing in 2014; 15AY, Anyang in 2015; 15ALE, Alaer in 2015; 16AY, Anyang in 2016. ^*c*^Xinluzao24. ^*d*^Lumianyan28.*

Most of the traits exhibited medium–high heritability across nine environments (**Supplementary Table S2**). Correlation analysis showed that significant or very significant positive correlations were observed between the trait pairs of FL–FS, FL–SI, FS–SI, FM–LP, FM–BW, and SI–BW; and significant negative correlations were observed between the pairs of FL–FM, FL–LP, FS–FM, FS–LP, BW–LP, and SI–LP. In addition, FL–BW showed a significant or very significant positive correlation in three environments, whereas no significant correlation was observed in the remaining six environments (**Table 2**).

**Table 2:** Correlation analysis between fiber quality and yield component traits in the RIL population.

Trait^a^	Environment^b^	FL	FS	FM	LP	BW
FS	13AY	0.271**				
	13QZ	0.510**				
	13LQ	0.139*				
	14AY	0.600**				
	14KEL	0.642**				
	14LQ	0.513**				
	15ALE	0.660**				
	15AY	0.466**				
	16AY	0.534**				
FM	13AY	–0.209**	–0.587**			
	13QZ	–0.226**	–0.576**			
	13LQ	–0.051	–0.348**			
	14AY	–0.417**	–0.486**			
	14KEL	–0.449**	–0.378**			
	14LQ	–0.413**	–0.463**			
	15ALE	–0.539**	–0.353**			
	15AY	–0.383**	–0.425**			
	16AY	–0.369**	–0.566**			
LP	13AY	–0.285**	–0.204**	0.366**		
	13QZ	–0.225**	–0.271**	0.454**		
	13LQ	–0.169*	–0.088	0.430**		
	14AY	–0.259**	–0.179**	0.220**		
	14KEL	–0.424**	–0.421**	0.351**		
	14LQ	–0.311**	–0.278**	0.360**		
	15ALE	–0.373**	–0.226**	0.352**		
	15AY	–0.149*	–0.088	0.044		
	16AY	–0.189**	–0.202**	0.336**		
BW	13AY	0.178**	–0.294**	0.352**	–0.197**	
	13QZ	0.006	–0.343**	0.543**	0.123	
	13LQ	0.127	–0.373**	0.544**	0.052	
	14AY	0.122	0.085	0.240**	–0.137*	
	14KEL	0.064	0.276**	–0.089	–0.352**	
	14LQ	0.060	–0.046	0.300**	–0.061	
	15ALE	0.237**	0.225**	–0.078	–0.146*	
	15AY	0.134*	–0.037	0.320**	–0.259**	
	16AY	–0.050	–0.199**	0.379**	–0.142*	
SI	13AY	0.284**	0.275**	–0.299**	–0.619**	0.324**
	13QZ	0.245**	0.250**	–0.159*	–0.467**	0.294**
	13LQ	0.187**	0.202**	–0.267**	–0.541**	0.094
	14AY	–	–	–	–	–
	14KEL	0.307**	0.453**	–0.102	–0.597**	0.565**
	14LQ	–	–	–	–	–
	15ALE	0.402**	0.418**	–0.179**	–0.470**	0.670**
	15AY	0.299**	0.363**	–0.182**	–0.309**	0.332**
	16AY	0.158*	0.336**	–0.192**	–0.256**	0.107

*^*a*^FL, fiber length; FS, fibers strength; FM, fiber micronaire; BW, weight; LP, lint percent; SI, seed index. ^*b*^13AY, Anyang in 2013; 13LQ, Linqing in 2013; 13QZ, Quzhou in 2013; 14AY, Anyang in 2014; 14KEL, Kerle in 2014; 14LQ, Linqing in 2014; 15AY, Anyang in 2015; 15ALE, Alaer in 2015; 16AY, Anyang in 2016. ^∗^Indicate significance at the 0.05 level. ^∗∗^Indicate significance at the 0.01 level.*

### Genetic Map Construction

**FIGURE 1:**
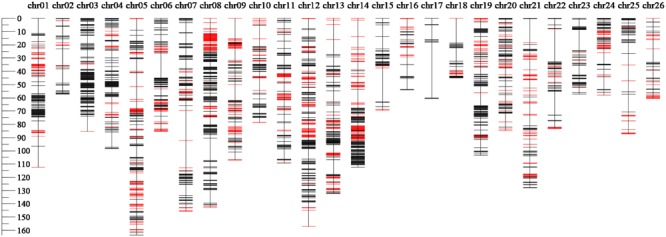
The genetic linkage map constructed by the SNP marker and SSR marker.

The genetic linkage map totally covered 2477.99 cM of the upland cotton genome with an average adjacent marker interval of 0.51 cM (**Figure 1** and **Table 3**). It contained 4851 markers, including 4729 SNP and 122 SSR loci, with uneven distributions in the A_t_ and D_t_ subgenomes as well as on 26 chromosomes. A total of 3300 markers were mapped in the A_t_ subgenome, covering a genetic distance of 1474.63 cM with an average adjacent marker interval of 0.45 cM. On the other hand, a total of 1551 markers were mapped in the D_t_ subgenome, covering a genetic distance of 1003.36 cM with an average adjacent marker interval of 0.65 cM. At the chromosome level, chr08 contained the maximum number of markers (481 markers), spanning a genetic distance of 142.55 cM with an average adjacent marker interval of 0.32 cM. chr17 contained the minimum number of markers (19 markers), spanning a total genetic distance of 60.60 cM with an average adjacent marker interval of 3.56 cM. Gap analysis revealed that there were 33 gaps (≥10 cM), of which 19 were in the A_t_ subgenome with the largest of 22.68 cM on chr07, whereas 14 were in the D_t_ subgenome with the largest of 42.23 cM on chr17. chr11, chr16, chr19, chr20 and chr24 had no gap larger than 10 cM.

**Table 3:** Detailed information of the genetic map.

Chr.	Number of SSRs	Number of SNPs	Total markers	Total distance (cM)	Average distance (cM)	Largest gap (cM)	Number of gaps (>10 cM)	Number of SDMs^a^	Percentage of SDMs (%)	SDR^b^ number	χ^2^-value	*P*-value
Chr01	2	234	236	112.38	0.49	15.83	2	90	38.14	3	2.17	0.46
Chr02	1	60	61	57.22	0.97	12.51	2	11	18.03	1	1.56	0.51
Chr03	6	304	310	85.26	0.28	11.59	1	3	0.97	0	0.75	0.56
Chr04	1	225	226	98.49	0.44	11.56	1	22	9.73	3	0.83	0.51
Chr05	15	279	294	163.46	0.57	10.44	1	138	46.94	10	3.93	0.22
Chr06	2	185	187	85.28	0.46	18.77	1	80	42.78	6	2.63	0.36
Chr07	11	220	231	145.52	0.66	22.68	2	44	19.05	5	2.09	0.38
Chr08	9	472	481	142.55	0.32	17.20	2	237	49.27	5	3.69	0.28
Chr09	3	222	225	107.03	0.49	15.63	2	96	42.67	8	2.83	0.25
Chr10	3	100	103	78.66	0.78	10.30	1	36	34.95	6	2.66	0.31
Chr11	2	203	205	109.13	0.57	7.68	0	97	47.32	6	3.06	0.22
Chr12	10	387	397	157.24	0.41	12.57	1	107	26.95	6	2.44	0.42
Chr13	7	337	344	132.40	0.40	11.47	3	100	29.07	7	2.70	0.45
A_t_	72	3228	3300	1474.63	0.45	22.68	19	1061	32.15	66	–	–
Chr14	4	315	319	112.26	0.36	12.16	2	161	50.47	9	3.37	0.25
Chr15	1	123	124	69.36	0.64	25.46	1	5	4.03	1	0.79	0.44
Chr16	4	51	55	53.91	0.98	8.83	0	26	47.27	2	2.70	0.26
Chr17	0	19	19	60.60	3.56	42.23	2	0	0.00	0	1.00	0.46
Chr18	1	57	58	45.05	0.79	18.45	2	9	15.52	1	1.39	0.47
Chr19	5	235	240	103.53	0.44	9.79	0	44	18.33	7	1.59	0.40
Chr20	12	133	145	84.74	0.59	6.37	0	32	22.07	4	1.70	0.36
Chr21	7	143	150	127.93	0.87	18.09	1	97	64.67	8	4.15	0.18
Chr22	1	92	93	83.17	0.92	24.89	2	32	34.41	5	2.43	0.31
Chr23	1	93	94	57.14	0.63	15.88	1	6	6.38	1	0.84	0.53
Chr24	7	106	113	57.89	0.52	9.26	0	40	35.40	3	2.46	0.28
Chr25	3	79	82	87.29	1.06	13.29	2	20	24.39	1	2.17	0.38
Chr26	4	55	59	60.49	1.06	10.09	1	30	50.85	2	3.64	0.23
D_t_	50	1501	1551	1003.36	0.65	42.23	14	502	32.37	44	–	–
Total	122	4729	4851	2477.99	0.51	42.23	33	1563	32.22	110	–	–

*^*a*^SDM, segregation distortion marker. ^*b*^SDR, segregation distortion region.*

### Segregation Distortion

There were a total of 1,563 SDMs (32.22%) (*P* < 0.05), which were unevenly distributed at both subgenome and chromosome levels (**Tables 3** and **Supplementary Table S3**). One thousand and sixty-one SDMs were found in the A_t_ subgenome, whereas 502 in the D_t_ subgenome. chr08 had the maximum number of SDMs of 237 (15.16% of total SDMs). The SDMs formed 110 SDRs, of which 66 were in the A_t_ subgenome whereas 44 in the D_t_ subgenome. chr05 contained the maximum number of SDRs of 10. There was no SDR in chr03 and chr17.

### Collinearity Analysis

**FIGURE 2:**
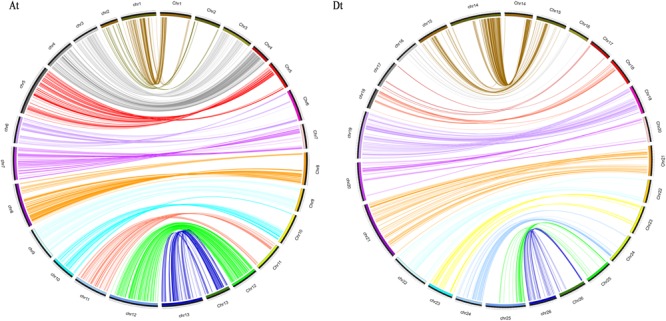
Collinearity between the genetic map (left) and the physical map (right). A_t_, collinearity of the A_t_ subgenome; D_t_, collinearity of the D_t_ subgenome.

The reliability of the genetic map was usually assessed by comparing it with the physical maps of the upland cotton (TM-1) reference genome (Zhang et al., 2015). The results of the collinear analysis are shown in **Figure 2**. The results revealed an overall good congruency between the linkage map and its physical one, while there also existed some discrepancies between the two on chr03, chr06, chr08, and chr13 in the A_t_ subgenome and on chr15, chr16, chr17, chr19, chr22, chr23, and chr26 in the D_t_ subgenome. The collinearity in subgenomes revealed that the A_t_ subgenome showed a better compatibility between the linkage and the physical maps than the D_t_ subgenome did.

### QTL Mapping for Fiber Quality Traits and Yield Components

**FIGURE 3:**
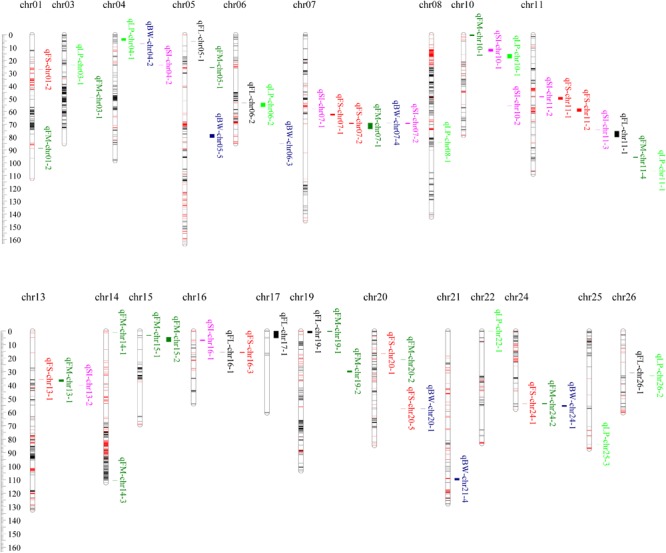
The annotation of the common candidate genes in GO analysis. **(A)** Fiber quality traits. **(B)** Yield components.

A total of 256 QTLs (**Supplementary Table S4**), 134 for fiber quality traits, and 122 for yield components, were identified across nine environments using the CIM algorithm, with 1.68–28.27% proportions of the phenotypic variance (PV) explained by each QTL. Fifty-seven stable QTLs (**Figure 3** and **Supplementary Table S4**) were identified in at least two environments, of which 32 were for fiber quality traits and 25 for yield components.

### Fiber Length

A total of 36 QTLs for FL were identified on 21 chromosomes except chr02, chr04, chr09, chr10, and chr25, among which 7 were stable (**Figure 3** and **Supplementary Table S4**). In these stable QTLs, qFL-chr17-1 was identified in three environments, and could explain 3.95–5.36% proportions of the observed PV. In its marker interval of TM53503–TM53577, there harbored 88 candidate genes. The stable QTLs, qFL-chr05-1, qFL-chr06-2, qFL-chr11-1, qFL-chr16-1, qFL-chr19-1, and qFL-chr26-1, could explain 12.13–13.83, 6.35–6.62, 5.15–9.41, 5.24–6.23, 4.65–5.07, and 4.56–5.59% proportions of the observed PVs, respectively. In their marker intervals of CICR0262, TM18200–TM18321, TM39956–TM39953, TM66757–NAU3563, TM57055–TM5 7082, and TM77259–TM77261, there harbored 2, 141, 15, 309, 65, and 1 candidate genes, respectively.

### Fiber Strength

Forty-six QTLs for FS were identified on 19 chromosomes except chr02, chr03, chr14, chr17, chr18, chr22, and chr23, among which 10 were stable (**Figure 3** and **Supplementary Table S4**). In these stable QTLs, qFS-chr07-2 was identified in all nine environments, and could explain 5.81–19.47% proportions of the observed PV. In its marker interval of DPL0852–DPL0757, eight candidate genes were harbored. qFS-chr16-3 was identified in five environments, and could explain 4.28–6.45% proportions of the observed PV. In its marker interval of SWU2707–DPL0492, 342 candidate genes were harbored. qFS-chr01-2 and qFS-chr20-5 were identified in three environments, and could explain 5.32–8.86 and 4.50–5.90% proportions of the observed PVs, respectively. In their marker intervals of TM379–TM404 and NAU4989–TM73152, 20 and 7 candidate genes, respectively, were harbored. qFS-chr07-1, qFS-chr11-1, qFS-chr11-2, qFS-chr13-1, qFS-chr20-1, and qFS-chr24-1 were identified in two environments, and could explain 5.97–6.21, 4.87–5.59, 5.26–7.21, 5.74–10.69, 2.91–8.18, and 5.11–5.43% proportions of the observed PVs, respectively. In their marker intervals of TM19848–TM19875, TM37826–TM37828, TM37897–TM37935, TM43230–TM43229, TM75088–TM75100, and TM67152–TM67146, 4, 1, 29, 1, 8, and 6 candidate genes, respectively, were harbored.

### Fiber Micronaire

Fifty-two QTLs for FM were identified on 21 chromosomes except chr02, chr12, chr17, chr23, and chr26, among which 15 were stable (**Figure 3** and **Supplementary Table S4**). In these stable QTLs, qFM-chr07-1 and qFM-chr13-1 were identified in six environments, and could explain 5.51–24.45 and 4.73–8.88% proportion of the observed PV, respectively. In their marker intervals of DPL0852–DPL0757 and TM43230–TM43241, 8 and 15 candidate genes, respectively, were harbored. qFM-chr01-2 was identified in five environments, and could explain 3.94–6.17% proportions of the observed PVs. In its marker interval, one marker of TM3451 was exclusively contained and two candidate genes were harbored. qFM-chr19-1 and qFM-chr19-2 were identified in four environments, and could explain 4.57–8.54 and 5.19–8.20% proportions of the observed PVs, respectively. In their marker intervals of TM57055–TM57057 and TM56813–TM56753, 4 and 161 candidate genes, respectively, were harbored. qFM-chr14-1, qFM-chr15-1, and qFM-chr24-2 were identified in three environments, and could explain 4.18–6.53, 4.45–5.35, and 4.25–4.69% proportions of the observed PVs, respectively. In their marker intervals of TM50241–TM50231, CGR5709–TM50087, and TM67152–TM67125, 13, 1, and 18 candidate genes, respectively, were harbored. qFM-chr03-1, qFM-chr05-1, qFM-chr10-1, qFM-chr11-4, qFM-chr14-3, qFM-chr15-2, and qFM-chr20-2 were identified in two environments, and could explain 3.89–5.18, 4.13–4.42, 4.66–5.16, 4.22–4.30, 4.52–4.54, 3.75–5.95, and 4.47–5.02% proportions of the observed PVs, respectively. In their marker intervals of TM7008–TM7102, TM10798–TM10805, TM33784–TM33813, TM39510–TM39490, TM52033–TM52031, TM50087–TM50082, and TM75041–TM75030, 125, 6, 100, 8, 1, 5, and 44 candidate genes, respectively, were harbored.

### Boll Weight

A total of 53 QTLs for BW were identified on 25 chromosomes except chr15, among which 7 were stable (**Figure 3** and **Supplementary Table S4**). In these stable QTLs, qBW-chr24-1 was identified in three environments, and could explain 4.13–6.99% proportions of the observed PVs. In its marker interval of TM67152–TM67127, 18 candidate genes were harbored. qBW-chr04-2, qBW-chr05-5, qBW-chr06-3, qBW-chr07-4, qBW-chr20-1, and qBW-chr21-4 were identified in two environments, and could explain 3.77–5.74, 4.28–6.42, 3.87–4.07, 7.62–8.08, 5.56–8.12, and 6.05–7.26% proportions of the observed PVs, respectively. In their marker intervals of TM9831–TM9827, TM10953–TM10979, TM14514–TM14509, DPL0852, NAU4989–CICR0002, and TM76018–TM75887, 6, 59, 23, 2, 7, and 119 candidate genes were harbored, respectively.

### Lint Percentage

A total of 39 QTLs for LP were identified on 20 chromosomes except chr02, chr12, chr15, chr17, chr23, and chr24, among which nine were stable (**Figure 3** and **Supplementary Table S4**). In these stable QTLs, qLP-chr10-1 was identified in five environments, and could explain 4.44–8.80% proportions of the observed PVs. In its marker interval of DPL0468–CGR5624, 148 candidate genes were harbored. qLP-chr04-1 was identified in four environments, and could explain 3.81–4.50% proportions of the observed PVs. In its marker interval of TM9862–TM9831, 217 candidate genes were harbored. qLP-chr26-2 was identified in three environments, and could explain 3.98–5.34% proportions of the observed PVs. In its marker interval of TM77259–TM77267, 3 candidate genes were harbored. qLP-chr03-1, qLP-chr06-2, qLP-chr08-1, qLP-chr11-1, qLP-chr22-1, and qLP-chr25-3 were identified in two environments, and could explain 2.69–2.83, 3.76–6.32, 4.43–6.02, 3.91–4.75, 3.61–4.26, and 4.77–7.64% proportions of the observed PVs, respectively. In their marker intervals of TM6006–TM6010, TM18161–TM18322, TM29470–TM29463, TM39443–TM39427, TM55461–TM55466, and TM63143–TM63142, 1, 141, 26, 12, 16, and 1 candidate genes, respectively, were harbored.

### Seed Index

A total of 30 QTLs for SI were identified on 16 chromosomes except chr01, chr14, chr15, chr18, chr21, chr22, chr23, chr24, chr25, and chr26, among which nine were stable (**Figure 3** and **Supplementary Table S4**). In these stable QTLs, qSI-chr07-2 was identified in five environments, which could explain 4.83–28.27% of the observed PVs. In its confidence interval of DPL0852–DPL0757, there harbored 8 candidate genes. qSI-chr16-1 was identified in four environments, which could explain 4.24–6.91% of the observed PVs. In its confidence interval of TM66717–TM66737, there harbored 19 candidate genes. qSI-chr10-1, qSI-chr10-2, and qSI-chr11-2 were identified in three environments, which could explain 6.67–7.83%, 4.28–6.50%, and 4.35–6.01% of the observed PVs, respectively. In their confidence intervals of DPL0468, TM36374–TM36487, and TM37826–TM37828, there harbored 2, 87, and 1 candidate genes, respectively. qSI-chr04-2, qSI-chr07-1, qSI-chr11-3, and qSI-chr13-2 were identified in two environments, which could explain 4.57–5.23%, 5.59–8.50%, 5.52–5.66%, and 3.37–5.29% of the observed PVs, respectively. In their confidence intervals of TM9702–TM9697, TM19691–TM19898, TM37970–TM39953, and TM43247–TM43263, there harbored 8, 39, 73, and 11 candidate genes, respectively.

### GWAS for Fiber Quality Traits and Yield Components

A total of 209 and 139 QTNs were identified by four multilocus GWAS methods to be associated with fiber quality and yield component traits, respectively, in the current study (**Supplementary Table S6**). Among these QTNs, 74 were simultaneously found by at least two algorithms or in two environments (**Supplementary Table S6**), each with 0.15–47.17% proportions of the observed PVs explained, and a total of 104 candidate genes were mined.

### Fiber Quality Traits

A total of 68, 65, and 76 QTNs were found to be associated with FL, FS, and FM, respectively, and the corresponding 110, 99, and 126 candidate genes were identified. In these QTNs, 11 for FL, 17 for FS, and 22 for FM were simultaneously associated by at least two algorithms or in two environments, and each could explain 0.15–29.10, 1.43–47.17, and 2.54–41.39% proportions of the observed PVs, respectively.

### Yield Components

A total of 51, 50, and 38 QTNs were found to be associated with BW, LP, and SI, respectively, and the corresponding 82, 83, and 65 candidate genes were identified. In these QTNs, 9 for BW, 5 for LP, and 10 for SI were simultaneously associated by at least two algorithms or in two environments, and each could explain 3.41–28.76, 3.00–22.49, and 1.21–38.73% proportions of the observed PVs, respectively.

### Candidate Genes Annotation

**FIGURE 4:**
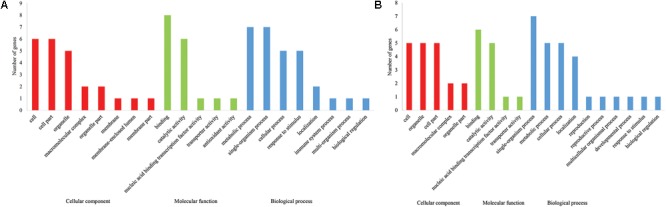
The chromosome-wise distribution of stable QTL for fiber quality traits and yield components.

A total of 2133 candidate genes, among which 621 were for FL, 426 for FS, 510 for FM, 234 for BW, 565 for LP, and 323 for SI, were identified from stable QTL (**Supplementary Table S5**), and 506 candidate genes, among which 110 for FL, 99 for FS, 126 for FM, 82 for BW, 83 for LP, and 65 for SI, were identified from GWAS (**Supplementary Table S6**). Annotation analysis of the 35 common genes from these two candidate gene pools revealed that 33 of them had annotation information, whereas 8 had unknown function (**Supplementary Table S7**). In the gene ontology (GO) analysis of the candidate gene for fiber quality (**Supplementary Table S8**), 24, 17, and 29 candidate genes were identified in the cellular component, molecular function, and biological process category, respectively. In the cellular component category, three main brackets of cell (six genes), cell part (six genes), and organelle (five genes) were enriched, whereas in the molecular function category, two main brackets of binding (eight genes) and catalytic activity (six genes), and in biological process category, four main brackets of metabolic process (seven genes), single-organism process (seven genes), cellular process (five genes), and response to stimulus (five genes) were, respectively, enriched (**Figure 4A**). In gene ontology (GO) analysis of the candidate gene for yield components (**Supplementary Table S10**), 19, 13, and 27 candidate genes were identified in the cellular component, molecular function, and biological process category, respectively. In the cellular component category, three main brackets of cell (five genes), organelle (five genes), and cell part (five genes) were enriched, whereas in the molecular function category, two main brackets of binding (six genes) and catalytic activity (five genes), and in the biological process category, four main brackets of single-organism process (seven genes), metabolic process (five genes), cellular process (five genes), and localization (four genes) were, respectively, enriched (**Figure 4B**). Kyoto encyclopedia of genes and genomes (KEGG) analysis indicated that six candidate genes for fiber quality were involved in 10 pathways and two candidate genes for yield were involved in six pathways (**Supplementary Tables S9, S11**).

## Discussion

### The High-Density Genetic Map Construction and Its Reliability

The development of high-throughput sequencing technology enabled its applications in genotyping the accessions of both natural populations for GWAS and segregating ones for map construction and QTL identification to be accumulated to agricultural important crops (Huang et al., 2010; Kump et al., 2011; Dhanapal et al., 2015; Zeng et al., 2017). SNPs provided abundant genetic variation loci at the genome level and much improved the genome coverage and marker saturation when they were applied to genetic map construction (Agarwal et al., 2008; Hulse-Kemp et al., 2015; Cai et al., 2017). At present, two sets of SNP arrays were developed for *Gossypium* (Hulse-Kemp et al., 2015; Cai et al., 2017). Different from the first set of CottonSNP63K arrays (Hulse-Kemp et al., 2015; Zhang Z. et al., 2017), which was developed by international consortium of several different studies (Hulse-Kemp et al., 2015), the CottonSNP80K array (Cai et al., 2017) was developed from the re-sequencing of 100 upland cotton cultivars and the TM-1 genome database (Zhang et al., 2015). Even though both sets were successfully applied in upland cotton linkage map construction and QTL identifications (Hulse-Kemp et al., 2015; Cai et al., 2017; Zhang Z. et al., 2017; Tan et al., 2018), the second set could have a higher genotyping accuracy, better coverage, and representative of *hirsutum* genome (Cai et al., 2017; Tan et al., 2018). In the current study, a linkage map was constructed mainly using SNP markers from the CottonSNP80K array in combination with SSR ones. The map spanned a total genetic length of 2477.99 cM, containing 122 SSR and 4729 SNP markers, with an average marker interval of 0.51 cM between adjacent markers. Compared with previous SSR maps (Shappley et al., 1998; Shen et al., 2005; Sun F.D. et al., 2012; Wang X. et al., 2015), the current map contained more markers and were more effective in map construction (Liu et al., 2015; Li et al., 2016; Zhang et al., 2016; Zhang Z. et al., 2017; Tan et al., 2018), and exhibited a high consistency with the genomic distribution of the SNP array, which demonstrated its representativeness in map construction (**Figure 2**; Cai et al., 2017).

The reliability of the genetic map is also estimated by gap size, collinearity, and segregation distortion analyses (**Figure 2** and **Table 3**). Although the development of SNP markers was based on the CottonSNP80K array, a few chromosomes still had a large gap or uneven distribution of makers (Li et al., 2016; Zhang Z. et al., 2017). Totally, there were 33 gaps larger than 10 cM, of which the largest one was of 42.23 cM on chr17 and there were only 19 markers mapped on it. The result of collinearity between the genetic map and the *G. hirsutum* (TM-1) reference genome indicated accuracy and quality of the map.

The segregation distortion is recognized as strong evolutionary force in the process of biological evolution (Taylor and Ingvarsson, 2003), which was also a common phenomenon in the study of genetic mapping (Shappley et al., 1998; Ulloa et al., 2002; Jamshed et al., 2016; Zhang et al., 2016; Tan et al., 2018). The current study observed that 32.22% of the total mapping markers were SDMs (*P* < 0.05). The maximum SDMs were on chr08, where there were 237 SDMs of the total 481 markers, forming five SDRs (**Figure 1**). This was in consistency with the SSR map constructed from the F_2_ population of the same parents of the current study (Kong et al., 2011). However, some studies observed an increase of the SDM ratio from F_2_ generation to the completion of RILs (Tan et al., 2018). This phenomenon was influenced by plenty of factors, including genetic drift (Shen et al., 2007) of mapping population, pollen tube competition, preferential fertilization of particular gametic genotypes, and others (Zhang et al., 2016; Zhang Z. et al., 2017; Tan et al., 2018). In the current study, some chromosomal uneven distribution of QTLs in SDR versus normal regions was also observed in chr01, chr06, chr07, chr10, chr16, chr19, and chr20. These facts implied an impact of the selections being imposed during the construction of the RIL population.

### Linkage and Association Analyses for Fiber Quality Traits and Yield Components

The QTLs detected in this study were compared with those in previous studies. As a result, 22 QTLs for FL, 25 QTLs for FS, 31 QTLs for FM, 36 QTLs for BW, 22 QTLs for LP, and 19 QTLs for SI in this study were coincided in the same physical regions of QTLs identified in previous studies (indicated with asterisks in **Supplementary Table S4**). The remaining could possibly be novel QTLs, of which 21 were stable ones, namely qFL-chr11-1, qFL-chr16-1, qFL-chr19-1, qFL-chr26-1, qFS-chr01-2, qFS-chr16-3, qFS-chr20-1, qFS-chr20-5, qFM-chr01-2, qFM-chr03-1, qFM-chr10-1, qFM-chr14-1, qFM-chr19-1, qBW-chr20-1, qLP-chr03-1, qLP-chr22-1, qLP-chr25-3, qLP-chr26-2, qSI-chr07-1, qSI-chr10-1, and qSI-chr16-1. Even though in the phenotypic evaluations of the population, the phenotypic differences between the two parents did not reach the significant level except that of FS, transgressive segregation in the RILs and significant differences among RILs indicated that the parents might harbor different favorable alleles for the target traits. QTL identification results well illustrated such presuppositions as these different favorable alleles contributed greatly to the similarity or nonsignificant differences between the two parents. These alleles could be addressed through map construction and detected in QTL identification. The high heritability of the target traits also enhanced the reliability of the QTL identification.

In addition, four multilocus GWAS algorithms were applied to the association of QTNs with the target traits, and their results were compared with the previous identified QTLs (Said et al., 2015). The results confirmed that quite a ratio of QTNs were coincided in the physical regions of the confidence intervals of reported QTLs in the database, namely 43 QTNs for FL, 44 QTNs for FS, 51 QTNs for FM, 40 QTNs for BW, 34 QTNs for LP, and 25 QTNs for SI (indicated with asterisks in **Supplementary Table S6**). The remaining QTNs could possibly be novel QTNs, of which 27 were associated by at least two algorithms or in two environments. These loci could be of great significance for cotton molecular-assisted breeding, particularly the loci of TM9941 and TM54893, which were identified both by multiple algorithms and in multiple environments for more than one target trait.

Based on linkage disequilibrium, GWAS is an effective genetic analysis method to dissect the genetic foundation of complex traits in plants in natural populations. The four multilocus GWAS algorithms provided promising alternatives in GWAS. Usually, GWAS needed a large panel size with sufficient marker polymorphism (Bodmer and Bonilla, 2008; Manolio et al., 2009), and was effective to identify major loci while ineffective to rare or polygenes (Asimit and Zeggini, 2010; Gibson, 2012) in the population. Linkage analysis in segregating populations could effectively eliminate the false-positive results, which was a built-in defect of GWAS in natural populations. But linkage analysis usually identified large DNA fragments, which made it difficult to further study the initial identification results. In the current study, both GWAS and linkage analysis were applied in the segregating RILs to study the correlations between genotypes and phenotypes. When comparing the results of GWAS to the QTLs of both previous studies (Said et al., 2015) and current study, common loci (genes) (**Supplementary Table S7**) demonstrated the effectiveness and feasibility of multilocus GWAS methods to address the correlation between genotypes and phenotypes in segregating RILs. Especially under the condition of increased marker density and improved genome coverage, the accuracy of QTN identification in GWAS would also increase. The increased accuracy probably rendered the application of GWAS in segregating population to have a higher effect on the observed PVs, sometimes even higher than that of QTL on the PVs in linkage analysis, which was usually low in natural populations.

### Congruency and Function Analysis of Candidate Genes

In this study, candidate genes were identified independently both from the physical region in the marker intervals of the QTLs, which were identified by CIM (Zeng, 1994) in WinQTL Cartographer 2.5 (Wang et al., 2012), and from the physical position of the QTNs, which were associated by multilocus GWAS algorithms. As the CIM algorithm gave not only the QTL position where the highest LOD value located, but also a marker interval of that QTL, the physical regions where the marker interval resided by QTL/QTN were used to search the candidate genes around the QTLs. To avoid redundant genes, the markers, which resided far away from the physical positions of the rest in the same confidence interval, were discarded for consideration of candidate gene searching. This increased the accuracy of the functional analysis of the candidate genes harbored in the confidence intervals of QTLs.

When comparing both candidate gene lists, even if they were not completely consistent, they still revealed a good congruency of candidate gene identification from both algorithms of QTL/QTN; namely, three congruent candidate genes for FL, seven for FS, nine for FM, five for BW, eight for LP, and nine for SI were identified (**Supplementary Table S7**). Further analysis of these candidate genes indicated that 1 for FL, 17 for FS, and 2 for FM (indicated with asterisks in **Supplementary Table S6**) were congruent with some previous reports (Huang et al., 2017; Sun et al., 2017). Two candidate genes, *Gh_D102255* (a protein kinase superfamily gene) and *Gh_A13G0187* (*actin 1* gene), which were for fiber quality, were also reported to participate in fiber elongation (Li et al., 2005; Huang et al., 2008). *Gh_A07G1730* and *Gh_D03G0236* belonged to a WD40 protein superfamily were mainly involved in yield formation in the current study, and might be related to a series of functions (Sun Q. et al., 2012; Gachomo et al., 2014). *Gh_D11G1653* (myb domain protein 6) functioned in BW formation, whereas reports indicated that several members of MYB family were involved in fiber development (Suo et al., 2003; Machado et al., 2009; Sun et al., 2015; Huang et al., 2016). Findings in the current study also indicated that some candidate genes could possibly be “pleiotropic,” namely *Gh_A07G1744* for FS, FM, and SI; *Gh_A07G1745* for FS and FM; *Gh_A07G1743* for BW and SI; and *Gh_D08G0430* for FM and BW. These candidate genes could be of great significance for further studies including functional gene cloning as well as cultivar development.

## Conclusion

The enriched high-density genetic map, which contained 4729 SNP and 122 SSR markers, spanned 2477.99 cM with a marker density of 0.51 cM between adjacent markers. A total of 134 QTLs for fiber quality traits and 122 for yield components were identified by the CIM, of which 57 are stable. A total of 209 and 139 QTNs for fiber quality traits and yield components were, respectively, associated by four multilocus GWAS algorithms, of which 74 QTNs were detected by at least two algorithms or in two environments. Comparing the candidate genes harbored in 57 stable QTLs with those associated with the QTN, 35 were found to be congruent, 4 of which were possibly “pleiotropic.” Results in the study could be promising for future breeding practices through MAS and candidate gene functional studies.

## Author Contributions

WG and YY initiated the research. WG, RL, and QC designed the experiments. RL, XX, and ZZ performed the molecular experiments. JG, JL, AL, HS, YS, QG, QL, MI, XD, SL, JP, LD, QZ, XJ, XZ, and AH conducted the phenotypic evaluations and collected the data from the field. RL, WG, YY, and HG performed the analysis. RL drafted the manuscript. YY and WG finalized the manuscript. All authors contributed in the interpretation of results and approved the final manuscript.

## Conflict of Interest Statement

The authors declare that the research was conducted in the absence of any commercial or financial relationships that could be construed as a potential conflict of interest.

## Footnotes

1 http://www.cottongen.org

2 http://www.cottondb.org

3 http://www.cottongen.org

4 http://mascotton.njau.edu.cn/info/1054/1118.htm
